# Diffusion Tensor Imaging Detects Microstructural Differences of Visual Pathway in Patients With Primary Open-Angle Glaucoma and Ocular Hypertension

**DOI:** 10.3389/fnhum.2018.00426

**Published:** 2018-10-22

**Authors:** Xiang-yuan Song, Zhen Puyang, Ai-hua Chen, Jin Zhao, Xiao-jiao Li, Ya-ying Chen, Wei-jun Tang, Yu-yan Zhang

**Affiliations:** ^1^Department of Ophthalmology, Huashan Hospital, Fudan University, Shanghai, China; ^2^Key Laboratory of Brain Functional Genomics (Ministry of Education), East China Normal University, Shanghai, China; ^3^Department of Radiology, Huashan Hospital, Fudan University, Shanghai, China

**Keywords:** primary open-angle glaucoma, ocular hypertension, diffusion tensor imaging, voxel-based analysis, central nerve system

## Abstract

Ocular hypertension (OHT), the common situation in adult patients in the outpatients, occurs ∼5% worldwide. However, there are still some practical problems in differentiation of OHT with early primary open-angle glaucoma (POAG) using current standard methods. Application of high resolution diffusion tensor imaging (DTI) enables us to the differentiate axonal architecture of visual pathway between POAG and OHT subjects. Among 32 POAG patients recruited (15 OHT and 14 control subjects), 62.5% of glaucoma were in early stage for the current study. All subjects underwent ophthalmological assessments with standard automated perimetry and optical coherence tomography (OCT). DTI was applied to measure fraction anisotropy (FA) and mean diffusivity (MD) of optic tract (OT), lateral geniculate body (LGN) and optic radiation (OR) using voxel-based analysis. Our data demonstrated that FA values of bilateral OR in POAG were significantly lower in the right or left than that of OHT patients (left OR: 0.51 ± 0.04 vs. 0.54 ± 0.03, *p* < 0.05; right OR: 0.51 ± 0.05 vs. 0.54 ± 0.03, *p* < 0.05). In right LGN, MD values were higher in POAG patients compared with OHT subjects (9.81 ± 1.45 vs. 8.23 ± 0.62, *p* < 0.05). However, no significant difference of all of the DTI parameters was observed between OHT and control subjects. DTI parameters in POAG patients were positively correlated with morphological and functional measurements (*p* < 0.05). Vertical cup to disc ratio (VCDR) was correlated with ipsilateral FA of OT (*p* < 0.05), ipsilateral MD of OT (*p* < 0.05), ipsilateral MD of LGN (*p* < 0.05), and contralateral MD of OT (*p* < 0.05). Mean deviation of visual field (MDVF) was correlated with ipsilateral FA of OT (*p* < 0.05), ipsilateral MD of OT (*p* < 0.05), and ipsilateral FA of LGN (*p* < 0.05). Our study demonstrated that DTI can differentiate POAG from OHT subjects in optic pathway, particularly in early POAG, and DTI parameters can quantify the progression of POAG.

## Introduction

Glaucoma, very common condition with approximately 79.6 million cases of blindness worldwide (Quigley and Broman, 2006), is rapidly increasing in the near future. Glaucoma is the name for a group of visual disorders which are characterized by the progressive loss of retinal ganglion cells (RGCs) and the degeneration of the optic nerve. Such changes lead to the progression of typical morphometric alterations that involve the optic nerve damages as well as visual field (VF) loss. Primary open-angle glaucoma (POAG) is the most prevalent type of glaucoma in the world (Weinreb and Khaw, 2004), which has a feature of progressive of optic nerve damage and loss of RGCs. Although there is a good correlation between high intraocular pressure (IOP) and the progression of POAG, its precise underlying pathophysiology is still unclear.

Ophthalmologists commonly encounter ocular hypertension (OHT) in outpatients, which occurs in 3.0–7.4% of all adults worldwide (Jacob et al., 1998). The definition of OHT is an IOP greater than two standard deviations from the mean of the normal ceiling value 21 mmHg with normal VF, and without evidence of glaucomatous optic disk changes (Sommer, 1989). Thus OHT is essentially different from POAG because of no RGCs damage.

The standard method of differential diagnosis for POAG and OHT is to use disk photography to measure thickness of retinal nerve fiber layer (RNFL) and visual functional testing (Plange et al., 2014). However, current method for RNFL measuring and visual functional testing is confined to assessing the state of RGCs. The thickness of RNFL can be detected by optical coherence tomography (OCT) to evaluate the degree of retinal damage in glaucoma, but it is limited in the interferences of age and myopia (Hoffmann et al., 2018; Tai et al., 2018). Functional testing like perimetry to measure VF reflects central nerve system (CNS) but cannot quantitate and specific injured part of CNS analysis. POAG is an insidious disease that is difficult to diagnose in its early stage, because routine VF tests cannot detect abnormalities until more than 50% of RGCs are damaged (Quigley, 1993; Casson et al., 2012).

In addition, VF value is quite frequently influenced by the degree of concentration of patients during examination. Thus there are some false positive and/or negative rates in OCT and/or VF examinations (Fingeret, 2012). Therefore, new method to identify POAG and OHT is important. Some features of POAG are similar to other CNS neurodegenerative disorders, such as ganglion cell loss and neuronal cell death like Alzheimer’s disease (Gupta and Yucel, 2007).

A series of studies demonstrate that pathological changes in the glaucomatous retina are associated with the visual pathway in CNS, especially in optic chiasm, lateral geniculate body (LGN) and optic radiation (OR) (Yucel et al., 2003; Kashiwagi et al., 2004; Gupta et al., 2006). Trans-synaptic degeneration is known as the degenerative spread of disease from affected neurons to healthy neurons through synaptic connections (Gupta and Yucel, 2007). It has been demonstrated that optic nerve axon is damaged earlier than RGCs in animal model (Buckingham et al., 2008), suggesting different pathological target(s) in the visual pathway is involved during the development of POAG or OHT.

Diffusion tensor imaging (DTI) is a quite novel technique based upon functional magnetic resonance imaging (fMRI) with non-invasive nature, which can observe upon white matter structure *in vivo* (Wieshmann et al., 2000), as the white matter tracts in the brain are composed of bundles of axons usually orientated in many different directions. Water diffusion occurs in any directions but preferentially occurs along a path parallel to the orientation of nerve fibers, because the cell membranes and other micro-structures serve as obstructions to water diffusion (Takahashi et al., 2000). This diffusion is called anisotropic and dependent on the integrity of white matter structures. Any disruption to the axons or changes in membrane permeability of the nerve fibers through demyelination alters the diffusion tensor indices (Horsfield and Jones, 2002). DTI is to measure mean diffusivity (MD) (a measurement of average molecular motion), and fractional anisotropy (FA) (a measurement of the regnant direction of diffusion) (Basser et al., 1994). FA reflects the main diffusivity directions of molecules, and MD reflects the extent of molecules’ diffusion out of axons. Therefore DTI can be used to measure modification of fiber microstructure of axons (Basser et al., 1994; Mori and Barker, 1999; Le Bihan et al., 2001). Decreased FA and increased MD are usually considered an indicator of axonal damage. Consequently the utilization of DTI is to identify the different nerve fiber structure within the scope of whole brain (Staempfli et al., 2007; Sherbondy et al., 2008).

Several studies have demonstrated that FA of the OR decreased and MD increased in Glaucoma patients (Garaci et al., 2009; El-Rafei et al., 2011; Tellouck et al., 2016). However, it is still unclear if DTI can be used for differential diagnosis of POAG and OHT.

Our aim was to investigate the differences of axonal architecture of the CNS between POAG and OHT subjects, using high resolution DTI to explore the neurodegenerative conditions.

## Materials and Methods

### Patients Recruitment

This observational cross-sectional study was approved by the Institutional Clinical Investigation Ethics Committee of Huashan hospital, Fudan University and fully complies with the Declaration of Helsinki on Biomedical Research. Written informed consent was obtained from all subjects prior to the study.

The participants of this study were divided into three categories, POAG patients, OHT subjects and control subjects. The POAG group included 32 patients (23 males and 9 females) with an average age of 52.2 ± 14.5 (mean ± standard deviation), and the OHT group was consisted of 15 subjects (8 males and 7 females) with average age 31.1 ± 12.4, while the control group was consisted of 14 subjects (9 males and 5 females) with average age of 40.6 ± 15.2 (Table 1). All participants were randomly selected from the outpatients within the department of ophthalmology of Huashan Hospital.

**Table 1:** Ophthalmologic measurements of control, OHT, and POAG groups.

	Control	OHT	POAG	*p*-value		
	*n* = 14	*n* = 15	*n* = 32	Control vs. OHT	Control vs. POAG	OHT vs. POAG
Age (years)	40.6(15.2)	31.1(12.4)	52.2(14.5)	0.248	0.009	<0.001
Male:Female	9:5	8:7	23:9	0.183	0.202	0.073
**Best corrected visual acuity SVAC**						
Right far	1.0(0.0)	1.0(0.1)	0.8(0.2)	0.448	<0.001	0.032
Left far	1.0(0.0)	0.9(0.1)	0.8(0.2)	0.271	0.003	0.191
Right near	1.0(0.0)	1.0(0.1)	0.5(0.3)	0.791	<0.001	0.001
Left near	1.0(0.0)	1.0(0.0)	0.5(0.3)	1.000	<0.001	0.001
Right IOP (mmHg)	15.4(0.8)	23.8(1.5)	16.8(4.6)	0.001	0.280	0.001
Left IOP (mmHg)	15.5(1.7)	23.6(0.9)	16.6(4.5)	0.001	0.377	0.001
Right central corneal thickness (μm)	526.0(22.8)	531.3(21.3)	525.4(32.7)	0.743	0.960	0.719
Left central corneal thickness (μm)	528.8(19.6)	513.5(56.5)	527.3(34.2)	0.435	0.917	0.487
Right axial length (mm)	24.1(1.3)	26.0(1.2)	26.1(1.6)	0.034	0.004	0.906
Left axial length(mm)	24.1(1.6)	26.0(1.5)	26.1(1.8)	0.074	0.013	0.882
Right mean sensitivity	30.5(1.5)	28.5(2.3)	21.8(5.7)	0.286	<0.001	<0.001
Left mean sensitivity	31.6(3.0)	28.2(2.6)	29.4(22.0)	0.618	0.720	0.823
Right loss variance	2.7(0.6)	4.9(2.7)	45.6(35.1)	0.839	<0.001	<0.001
Left loss variance	2.6(0.3)	7.9(5.7)	37.2(33.5)	0.606	<0.001	0.001
Right mean deviation	0.7(0.5)	−0.4(2.1)	6.5(6.2)	0.564	0.001	<0.001
Left mean deviation	0.5(0.3)	0.1(2.8)	5.0(5.3)	0.822	0.003	0.001
Right RNFL thickness (μm)	98.6(11.6)	96.7(7.0)	72.3(15.2)	0.717	<0.001	<0.001
Left RNFL thickness (μm)	96.6(11.5)	91.3(9.2)	75.8(10.7)	0.221	<0.001	<0.001
Right VCDR	0.4(0.2)	0.5(0.2)	0.8(0.1)	0.069	<0.001	<0.001
Left VCDR	0.4(0.2)	0.6(0.1)	0.7(0.1)	0.003	<0.001	<0.001

*Data were presented as mean(SD). The population distribution by gender was compared with Chi-square test, while others were analyzed by ANOVA with LSD test.*

### Ophthalmologic Examination

All participants in the current study underwent a complete ophthalmological examination, including best-corrected visual acuity, IOP, slit lamp biomicroscopy, and optic disk examination by funduscopy. Central corneal thickness was assessed by non-contact specular microscopy, and RNFL thickness was measured using spectral-domain optical coherence (SD-OCT; Cirrus, Carl Zeiss Meditec, Inc.). All images were acquired by the trained technicians in masked fashion. All participants underwent VF testing using Octopus 101 (Haag-Streit, Inc., Bern, Switzerland, dynamic strategy). To improve the reliability of our results, the quality standard was as follows: false-positive errors was <15%, false-negative errors was <15%, and loss fixations was <20%. In consideration of artifacts, it was excluded from the subjects with eyelid or rim artifacts, fatigue effects, inappropriate fixation or inattention, VFs examination.

Primary open-angle glaucoma patients are defined as follows (Prum et al., 2016): (1) monocular or binocular glaucomatous VF defects (worse than +2 db) or defects involving the nerve fiber layer. (2) The presence of glaucomatous optic disk, with a cup-to-disk ratio greater than 0.5, a thinning rim, notching, or excavation. (3) The anterior chamber angle must be opened.

The patients who met the conditions of 1 and 3, or 2 and 3 mentioned above were included in the glaucoma group. In addition, stage of severity of glaucoma was classified according to the Hodapp-Parrish-Anderson classification (Maeda et al., 1994). The different stages are as follows:

Stage 0: no or minimal defect
Stage 1: MD ≥−6.0 dB (early defect)
Stage 2: −12.0 ≤ MD ≤ −6.0 dB (moderate defect)
Stage 3: −20 ≤ MD ≤ −12.0 dB (advanced defect)
Stage 4: MD ≤ −20.0 dB (severe defect)
Stage 5: end-stage disease

The subjects whose IOP > 21 mmHg with none functional and organic damages to their vision and outpatient follow-up for 1 year without VF change were selected for the OHT group. All control subjects underwent ophthalmologic examinations in the same way as the patients were included as POAG and OHT groups. The exclusion criteria for all subjects included: (1) Those suffering from other ophthalmologic diseases which can affect the VF. (2) Those who cannot undergo ophthalmologic or MRI examinations. (3) Those suffering from neurological diseases such as cerebral infarction, mental retardation or craniocerebral trauma.

Age, mean defect, RNFL thickness (gauged by OCT) of all subjects were followed normal distribution.

### MRI Acquisition

All subjects involved in this study were scanned by a 3 Tesla scanner (Siemens Magnetom Verio 3T). A dedicated 12-channel head coil was equipped for radiofrequency signal transmission and reception. The imaging protocol included T1-weighted sequence, T2-weighted sequence, and three-dimensional magnetization prepared rapid acquisition gradient echo sequence (3D-MPRAGE). The acquisition parameters applied were: TR = 2300 ms, TE = 2.98 ms, FOV = 256 mm, matrix = 256 × 256, slice thickness = 1 mm isovoxel, number of slices = 176, and the image scan time was 5 min 3 s. The DTI parameters which were used to cover the whole brain were as follows: TR = 8500 ms, TE = 87 ms, FOV = 23 mm, matrix = 128 × 128, slice thickness = 3.0 mm, number of slices = 46, 20 directions, diffusion-weighted factor, *b* = 1000 s/mm^2^ and image scan time of 6 min 24 s.

### Image Processing

The processing of DTI was performed using the FMRIB’s diffusion toolbox (FDT) of FMRIB’s free software library (FSL). Firstly, the eddy current distortions of the DTI images were corrected by the program eddy correct; then the parameter maps of FA and MD were obtained by the program dtifit.

**FIGURE 1:**
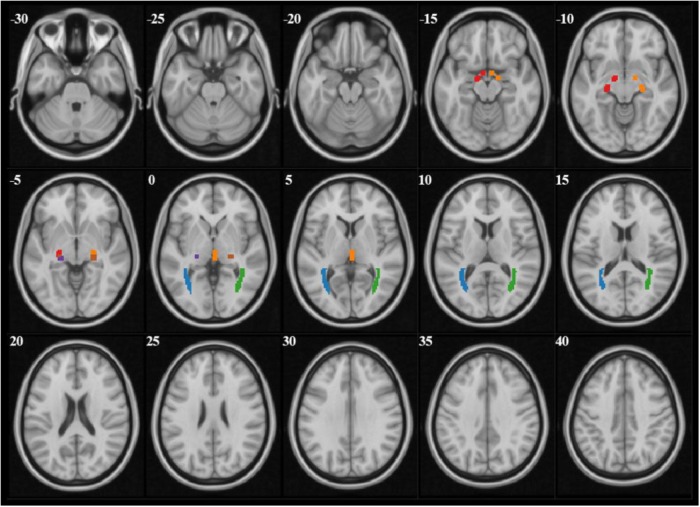
The mask files of each ROI demonstrated on *b* = 0 images. Red and orange manifested right and left OT, purple and brown manifested right and left LGN, blue and green manifested right and left OR.

The analysis of diffusion parameters was performed using SPM8 toolbox on Matlab, 2012a workstation. For each subject, *b* = 0 images were first co-registered to the 3D-MPRAGE images by the program coregister; then the 3D-MPRAGE images of all subjects were normalized to the Montreal Neurological Institute (MNI) standard space by the program segment and the normalized parameters were obtained, after that the FA and MD maps were also normalized to the MNI standard space with the normalized parameters which were obtained from last step by the program normalize. According to the coordinate of MNI space, the mask file of 6 seed-regions of interests (ROIs) (bilateral OT, LGN, and OR) were made by program WFU PickAtlas (Figure 1). Finally the FA and MD values corresponded with ROIs were extract from FA and MD maps.

### Statistical Analysis

In order to take account of the influence of age, an analysis of covariance (ANCOVA) with age as covariate was applied to compare DTI parameters of POAG, OHT, and control groups. The least square difference (LSD) test was used for *post hoc* analyses. Associations between DTI parameters with ophthalmologic measurements were described by Pearson’s correlation coefficients. Data were presented as mean ± standard deviation unless otherwise specified.

## Results

### Demographic and Ophthalmologic Characteristics

All demographic information and ophthalmologic data of POAG, OHT, and control subjects were as shown in Table 1. In the current study, according to Hodapp-Parrish-Anderson classification, there was 62.5, 25, or 12.5% of glaucoma patients were in early, moderate stage, or advanced stage, respectively. Age of POAG group was significantly older than that of the control (52.5 ± 14.5 vs. 40.6 ± 15.2) or OHT (52.5 ± 14.5 vs. 31.1 ± 12.4) groups with *p* < 0.05 among them all (ANOVA with LSD *post hoc* test). Thus the interference of age was taken into consideration.

### Comparisons of DTI Measurements in Visual Pathway

To examine the integrity of white matter through intracranial visual pathway, two DTI parameters, i.e., FA and MD of 6 ROIs (bilateral OT, LGN, and OR) were analyzed among POAG, OHT, and control groups (Table 2).

**Table 2:** Comparisons of DTI parameters among POAG, OHT, and control subjects.

ROI	DTI parameters	Group			*P*-value			
		POAG	OHT	Control	POAG vs. control	OHT vs. control	POAG vs. OHT	*P* covariant
Left OT	FA	0.39 ± 0.07	0.41 ± 0.04	0.38 ± 0.03	0.703	0.247	0.394	0.673
	MD, 10^−4^ mm^2^/s	12.11 ± 1.69	10.68 ± 1.08	10.38 ± 1.85	0.002^∗^	0.306	0.081	0.636
Right OT	FA	0.36 ± 0.09	0.37 ± 0.07	0.35 ± 0.04	0.915	0.626	0.686	0.592
	MD, 10^−4^ mm^2^/s	13.72 ± 2.93	12.38 ± 2.74	12.13 ± 2.20	0.135	0.649	0.384	0.962
Left LGN	FA	0.42 ± 0.06	0.43 ± 0.07	0.44 ± 0.05	0.403	0.179	0.521	0.509
	MD, 10^−4^ mm^2^/s	8.54 ± 0.97	7.98 ± 0.94	7.90 ± 0.63	0.024^∗^	0.542	0.159	0.944
Right LGN	FA	0.41 ± 0.07	0.42 ± 0.06	0.44 ± 0.07	0.123	0.094	0.702	0.752
	MD, 10^−4^ mm^2^/s	9.18 ± 1.45	8.23 ± 0.62	7.80 ± 0.64	0.001^∗^	0.284	0.046^∗^	0.967
Left OR	FA	0.51 ± 0.04	0.54 ± 0.03	0.53 ± 0.03	0.033^∗^	0.664	0.020^∗^	0.314
	MD, 10^−4^ mm^2^/s	8.96 ± 1.08	8.39 ± 0.64	8.38 ± 0.54	0.123	0.816	0.255	0.364
Right OR	FA	0.51 ± 0.05	0.54 ± 0.03	0.53 ± 0.04	0.047^∗^	0.489	0.028^∗^	0.315
	MD, 10^−4^ mm^2^/s	8.73 ± 0.95	8.25 ± 0.38	8.38 ± 0.52	0.26	0.698	0.164	0.495

*Data were presented as mean ± SD. ^∗^P-value < 0.05, analysis of covariance.*

Compared with control group, FA values of bilateral OR in POAG patients were significantly lower (left OR: 0.51 ± 0.04 vs. 0.53 ± 0.03, *p* < 0.05; right OR: 0.51 ± 0.05 vs. 0.53 ± 0.04, *p* < 0.05; ANCOVA with LSD *post hoc* test); MD values of POAG patients were higher in bilateral LGN (left LGN: 8.54 ± 0.97 vs. 7.90 ± 0.63, *p* < 0.05; right LGN: 9.18 ± 1.45 vs. 7.80 ± 0.64, *p* < 0.05) and in left OT (12.11 ± 1.69 vs. 10.38 ± 1.85, *p* < 0.05).

Compared with OHT subjects, FA values of bilateral OR of patients with POAG were significantly lower (left OR: 0.51 ± 0.04 vs. 0.54 ± 0.03, *p* < 0.05; right OR: 0.51 ± 0.05 vs. 0.54 ± 0.03, *p* < 0.05). MD values of POAG patients were higher in right LGN compared with the OHT (Right LGN: 9.81 ± 1.45 vs. 8.23 ± 0.62, *p* < 0.05).

There was no difference in terms of FA or MD between OHT and control groups. The influence of age was all excluded aforementioned as covariant.

Together, DTI results demonstrated that POAG patients had different FA and MD in certain areas of visual pathway compared to OHT or control groups; whereas DTI imaging was unable to detect obvious discrepancy between OHT and control groups.

### Correlations of DTI Parameters With the Severity of the POAG Patients

To verify the feasibility of DTI parameters in evaluating POAG progression, we applied correlation analysis between DTI parameters and ophthalmologic measurements which represented the severity of POAG (Table 3). Vertical cup to disc ratio (VCDR) and thickness of RNFL were measured to characterize the morphological changes in POAG patients, and mean deviation of visual field (MDVF) was measured to examine functional changes. The correlations of ophthalmologic parameters with DTI parameters were separated into ipsilateral (right eye-right ROIs and left eye-left optic ROIs) and contralateral (right-left and left-right) sides.

**Table 3:** Correlation of DTI parameters with morphological or functional measurements in POAG patients.

ROI	DTI parameter	VCDR		RNFL thickness		MDVF	
		r	P	r	P	r	P
**Ipsilateral**							
OT	FA	−0.33	0.020^∗^	0.14	0.313	−0.32	0.014^∗^
	MD	0.39	0.005^∗^	−0.25	0.059	0.30	0.023^∗^
LGN	FA	−0.25	0.072	0.13	0.337	−0.29	0.027^∗^
	MD	0.32	0.024^∗^	−0.15	0.278	0.18	0.171
OR	FA	0.01	0.927	−0.01	0.973	−0.02	0.912
	MD	0.03	0.858	−0.08	0.567	−0.10	0.442
**Contralateral**							
OT	FA	−0.22	0.128	−0.04	0.753	−0.19	0.148
	MD	0.30	0.033^∗^	0.07	0.608	0.14	0.290
LGN	FA	−0.15	0.284	−0.04	0.761	−0.19	0.147
	MD	0.21	0.131	0.07	0.610	−0.01	0.953
OR	FA	−0.09	0.526	0.04	0.746	−0.12	0.381
	MD	0.10	0.473	−0.12	0.386	0.02	0.897

*r, Pearson’s correlation coefficient. ^∗^P-value < 0.05.*

**FIGURE 2:**
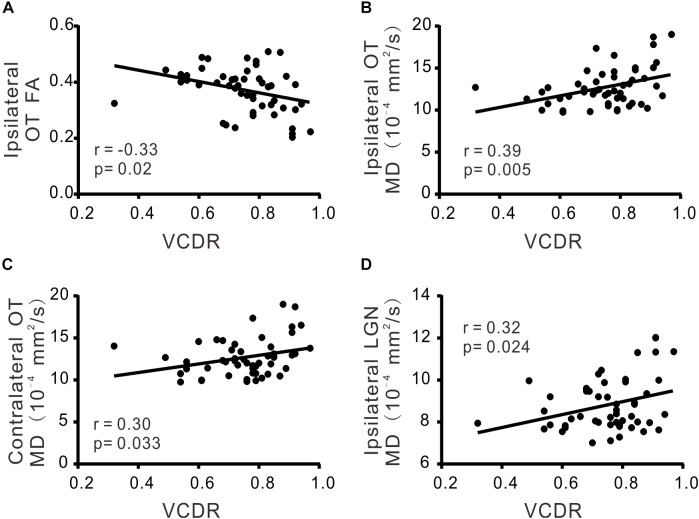
The correlations of DTI parameters and VCDR. FA **(A)** and MD **(B)** in ipsilateral OT, MD in contralateral OT **(C)**, MD in ipsilateral LGN **(D)** were significantly correlated with VCDR.

**FIGURE 3:**
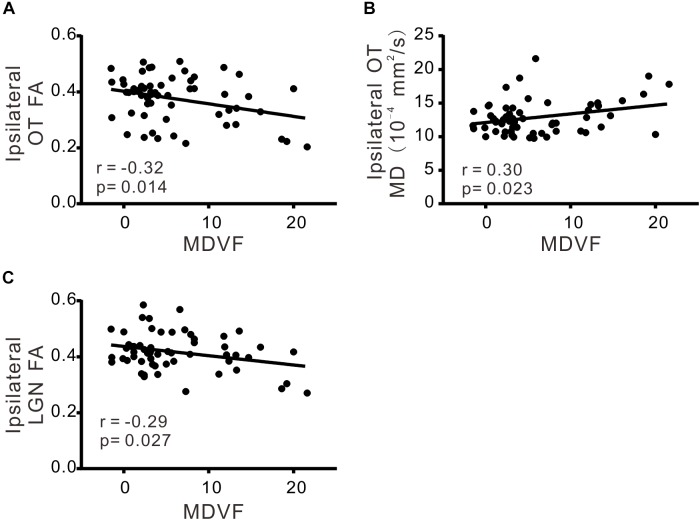
The correlations of DTI parameters and MDVF. FA **(A)** and MD **(B)** in ipsilateral OT, FA in ipsilateral LGN **(C)** were significantly correlated with MDVF.

There was significant correlation between VCDR and ipsilateral FA of OT (*r* = −0.33; *p* < 0.05), ipsilateral MD of OT (*r* = 0.39; *p* < 0.05), ipsilateral MD of LGN (*r* = 0.32; *p* < 0.05), or contralateral MD of OT (*r* = 0.30; *p* < 0.05), respectively (Table 3 and Figure 2). Moreover MDVF was significantly correlated with ipsilateral FA of OT (*r* = −0.32; *p* < 0.05), ipsilateral MD of OT (*r* = 0.30; *p* < 0.05), or ipsilateral FA of LGN (*r* = −0.29; *p* < 0.05), respectively (Table 3 and Figure 3). However, there was no significant correlation between RNFL thickness and DTI parameters.

## Discussion

Our current study mainly demonstrated microstructural differences of ROIs (OT, OR, and LGN) between POAG and OHT, as evaluated by lower FA in bilateral OR and higher MD in right LGN using DTI. It was further verified DTI parameters (FA and MD) were correlated with structural impairment and functional decline in POAG patients.

### Diagnose OHT and POAG With DTI

OHT subjects have an IOP > 21 mmHg without VF loss and without glaucomatous optic disk changes. The differences between OHT and POAG are based on whether there is progressive of optic nerve damage and loss of RGCs. However, it is difficult to differentiate of POAG in early stage and OHT, because POAG is frequently misdiagnosed without obvious VF defection. Increasing evidence demonstrates that POAG, a neurodegenerative disease, could affect intracerebral optic pathway in its early stage (Yucel et al., 2003; Kashiwagi et al., 2004; Gupta et al., 2006; Gupta and Yucel, 2007).

The advantages of DTI enable us to compare the integrity of white matter tracts within bilateral OT, LGN, and OR between POAG and OHT, which has not been previously reported in literature. There was no significant difference between OHT and control group in all six regions (bilateral OT, LGN, and OR) as expected, while POAG patients exhibited significant microstructural changes. These evidences of differentiation between POAG and OHT are helpful for ophthalmologists to decide whether to apply medical or surgical treatments when facing with a patient whose IOP is greater than 21 mmHg without experiencing VF loss.

Primary open-angle glaucoma is a group of neurodegenerative diseases involving death of retinal ganglion RGCs and damage of optic nerve (Gupta et al., 2006). Studies on POAG in human and animals reveal that white matter of components in the optic pathway is affected, reflecting by FA and MD measurements using DTI technique. For instance, a decrease in FA and an increase in MD are observed in the optic nerve in glaucomatous rats (Hui et al., 2007). Garaci et al. (2009) demonstrate that decreased FA values and increased MD in POAG group, respectively (El-Rafei et al., 2011; Tellouck et al., 2016). This is consistent with our current study, showing decreased FA in bilateral OR and increased MD in left OT and bilateral LGN when compared to control group, which is supported by Tellouck et al. (2016) study. In our current study, POAG group had lower FA in bilateral OR and higher MD in right LGN than OHT group. This is in line with our current finding, showing FA decreased and MD increased in POAG than control group. These changes are most likely resulted from the disruption in optic nerve fiber in POAG patients. Interestingly, there was difference in right LGN other than left LGN. In our study, POAG had different average MDVF in both eyes, which was 6.5 in right and 5.0 in left. That is to say CNS damage may in different stage in two side, and right side had slight severer than left side.

Francesca et al report that FA is a more reliable and sensitive measurement of neurodegeneration than MD (Bolacchi et al., 2012). This is supporting our current study, showing that FA was decreased in bilateral OR from POAG compared to OHT group. Interestingly, none statistical difference of MD in OR was observed in our study, suggesting MD was less sensitive than FA in the differential diagnosis between POAG and OHT. It may also be due to relatively smaller in sample size in the current study. Besides, there was relatively higher proportion of early stage POAG, which may have slighter damage than severe glaucoma. Both of the two reasons may compromise the statistics difference. Our study firstly compared visual pathway difference using DTI between POAG and OHT, perhaps we need more researches to testify the results. Similarly, Tellouck et al. (2016) find decreased FA in bilateral OR and none MD change in their study, which has 70% POAG in early stage.

However, there was no significant difference between OHT subjects and controls in all ROIs (bilateral OT, LGN, and OR). Usually, decreased FA and increased MD are considered an indicator of axonal damage (Le Bihan et al., 2001). DTI (FA and MD), quantify directionally diffusion of water molecules along white matter tracts, can detect axonal loss, demyelination, edema, and gliosis (Song et al., 2003; Owler et al., 2006; Budde et al., 2011). Thus our results confirm no optic nerve damage in OHT subjects although they had higher IOP. It is noted that most POAG (62.5%) patients were in early stage in our current study, and it was demonstrated that DTI seems to be a reliable approach in differentiation diagnosis of early stage POAG and OHT.

### Correlation of DTI Parameters and POAG Severity

Most of the significant correlation was on MD or FA of OT with ipsilateral VCDR or VF defection. Our explanation is that because OT is a part of optic nerve which is the axon of RGCs, alteration in OT probably is the most relevant to the loss of RGCs, leading to structural and/or visual functional degeneration in POAG.

In addition there was correlation between MD of OT and contralateral VCDR, but other significant associations from the different regions were in ipsilateral part, which is consistent with the study by Tellouck et al. (2016). Because the ROIs (OT, LGN, and OR) we chose had 50% primary nerve fibers from contralateral eye, we expect some similar associations of POAG between severity parameter and DTI parameter bilaterally. However, our findings demonstrated the POAG disease severity was mainly associated in ipsilateral visual pathway. This may be due to more vulnerable of the temporal and temporal-inferior sides of the optic nerve head in POAG (Tan et al., 2009; Strouthidis et al., 2011; Yang et al., 2013), resulting in a more serious atrophy on ipsilateral optic pathway structure.

Interestingly, VCDR was more closely associated with ipsilateral diffusion parameters than MDVF in our study. As mentioned previously, most of our POAG patients were in early stage, subsequently structural change was more sensitive than visual function in early POAG (Miglior et al., 1996). However, there was no significant association between the thickness of RNFL and diffusion parameters. We speculate that big variation of axial length influenced the thickness of RNFL (Patel et al., 2018).

### Limitations

We acknowledge that there are some limitations. Relatively small sample size may result in a lack of significant difference in MD, and this may have limited our statistical power to detect significant differences in DTI parameters between the POAG and OHT groups. In the process of recruiting subjects, the axial length of these groups was not in a same standard. The axial length of POAG patients was about 26.1 mm, which was beyond normal length. It probably compromised the correlation between the thickness of RNFL and diffusion parameters.

## Conclusion

As an insidious disease, POAG is difficult to diagnosis in its early stage. Our study demonstrated that DTI could be a useful tool to differentiate OHT and early POAG. DTI parameters could also serve as quantifiable assessments for the progression of POAG.

## Author Contributions

Y-yZ, W-jT, X-yS, and ZP designed the research. Y-yZ, W-jT, and A-hC provided the materials. X-yS, ZP, JZ, and W-jT performed the experiments and data analysis. ZP, X-yS, and W-jT prepared the figures and wrote the manuscript. X-yS, ZP, A-hC, JZ, X-jL, Y-yC, W-jT, and Y-yZ edited and approved the manuscript.

## Conflict of Interest Statement

The authors declare that the research was conducted in the absence of any commercial or financial relationships that could be construed as a potential conflict of interest.
